# Therapeutic pluralism and the politics of disclosure: breast cancer patients' experiences in public healthcare

**DOI:** 10.4314/ahs.v22i4.11

**Published:** 2022-12

**Authors:** Jennifer Nyawira Githaiga, Leslie Swartz

**Affiliations:** 1 University of Cape Town, Division of Social & Behavioural Sciences, School of Public Health; 2 Stellenbosch University, Department of Psychology

**Keywords:** Breast cancer, alternative medicine, South Africa

## Abstract

**Background:**

Despite the widespread utilisation of complementary and or alternative medicine (CAM) by breast cancer patients in low-and-middle-income countries, few disclose CAM use to their physicians.

**Objective:**

This study examines disclosure CAM use among a small sample of women attending a breast cancer clinic in a public health hospital in the Western Cape, South Africa.

**Methods:**

An Interpretative Phenomenological Analysis (IPA) approach was utilised in this study. Semi-structured in-depth interviews were conducted with a convenience sample of 17 women attending a breast clinic in a public hospital.

**Results:**

Non-disclosure of CAM use was attributed to minimal time for patient-doctor interactions due to resource constraints in public health facilities and the superior status accorded to biomedical doctors' superior knowledge, associated with paternalism, leading to patients' fear of reproach for using CAM. Consequently, disclosure of CAM only occurred in instances where it was deemed an absolute necessity.

**Conclusion:**

Considering the reality of an overstretched public healthcare system, what may be possible is an active attempt to communicate to patients that doctors are aware that patients may use a range of resources (such as CAM), which is their right, and further, recommend patient disclosure of CAM use to their doctors, because of the possibility of drug interactions and other potentially negative effects.

## Introduction

Complementary and or alternative medicine (CAM) for breast cancer treatment is widely documented in Low-and Middle-Income Countries (LMICs) in settings where: healthcare is not subsidised^1^–^3^; there are limited facilities^4^,^5^, and dissatisfaction with healthcare services^6^–^8^; cultural beliefs support the use of CAM^1^,^3^,^7^. Despite the widespread use of CAMs, relatively few patients disclose their use of CAMs to personnel in conventional healthcare facilities 9. Studies in several countries suggest that patients viewed such disclosure as unnecessary, or they anticipated negative reactions to disclosure 4, 10, 11. Disclosure is however in the best interests of patients as there may be risks associated with using CAMs alongside biomedical therapies^10^–^12^. In the South African context CAM use has been reported in the context of communicable diseases, and specifically HIV^13^–^15^, and non-communicable diseases (NCDs)^16^,^17^, including cancer 18–20. The cited articles report on CAM use either in the complementary sense of medical pluralism or as an alternative to biomedical treatment options, with some scholars positing an association between CAM use and sociocultural health-related beliefs 14, 16–18. Three pertinent themes featured in CAM studies in South Africa are CAM use as a cause of delays in assessing treatment 14, 18, CAM use and interruption in adherence to biomedical treatment modalities 14, 20 and concerns about potentially negative interactions between CAMS and biomedical treatments 16, 17. In discussions of these three themes, disclosure of CAM use was mentioned from the perspectives of: patients, some of whom viewed disclosure as unnecessary^17^, and; health practitioners, some of whom did not expect patients to be using CAMs and, as such, did not inquire about this from patients^17^, while others argued that health practitioners who support medical pluralism refrain from disclosure of referrals to CAM practitioners in fear of disapproval from colleagues who subscribe solely to biomedical treatments^14^.

Against this background, scholars advocated for more research on the prevalence of CAM use among patients 16, 17, including motivations for CAM use 15, and the need to improve physician-patient communication around CAM use 17, 20 to facilitate informed choices among patients 20. In the specific context of breast cancer patients, CAM use was mentioned in the context of socio-cultural beliefs and potential delays in help-seeking but not explored in detail 18, 19. We sought to contribute to the existing gaps by examining disclosure of CAM use among women attending a breast cancer clinic in a public health hospital in the Western Cape, South Africa. CAM use was explored in the context of women's narrative accounts of their lived experiences of the phenomenon of breast cancer in its entirety, of which CAM use was one of several aspects of some (not all) women's experiences.

## Methods

We utilised Interpretative Phenomenological Analysis (IPA), a qualitative research approach which investigates lived experiences of individuals and the meanings they ascribe to their experiences 21–23. IPA posits that while individuals interact with mundane experiences without being explicitly aware of these, when individual encounter significant life events, they begin to consciously attempt to make sense of such experiences 22. IPA seeks to unpack how individuals make sense of significant life experiences within their social contexts using individuals' subjective narrative accounts of their experiences as the primary sources of data. We deemed this a suitable approach to investigate women's lived experiences of breast cancer, which included exploring experiences of CAM use, with a focus on individual subjective accounts. This is consistent with IPA's idiographic component characterised by a small sample size to allow detailed exploration of each individual's nuanced experiences and how each one makes sense of their experience of the phenomenon under study 21–23.

A convenience sample of women patients at a breast clinic at a public tertiary hospital in the Western Cape. Seventeen participants were included in the study (see Table 1). Interviews were conducted by the lead author and three postgraduate researchers fluent in both English and Afrikaans. The lead author and co-author are both qualified health professionals and academics. Two of the postgraduate researchers were qualified health professionals, one of whom was enrolled as a postgraduate research psychology student at the time of this study. The third researcher was also a postgraduate research psychology student. The interview team were introduced to the clinic staff prior to the start of data collection. The clinic nurses, who had regular and direct access with breast cancer patients, explained the study to patients in the waiting area and then linked the researchers with volunteer participants. All the interviewers were required to wear their university identification cards at all times while on site, to distinguish themselves from clinical staff, and introduced themselves as part of a university research team conducting a study on breast cancer experiences. This clarification of our positionality was important based on previous research experience in similar public hospital set ups where researchers may be mistaken for medical staff particularly in the context of shared spaces, for example, when conducting a research interview in a medial consultation room when this is the only private space available. In this context, and in keeping with IPA's acknowledgement of the role of reflexivity in making sense of peoples' lived experiences and specifically how one's preconceptions shape the research process 21, 22, reflexivity necessitated recognising, rather than bracketing the reality the that (a) though, for purposes of the study, research team members' designated role was ‘research interviewer’, each one entered the research space bearing various simultaneous positionalities [for example, health professional, researcher, student, caregiver of a close family member or friend with breast cancer, woman, mother, daughter, wife] (b) these positionalities shaped interview interactions with participants and subsequent making sense of arising research data. Each interviewer documented reflexive aspects of their interactions with each participant, as detailed in a subsequent paragraph describing the audit trail as part of ensuring rigour in the study. Semi-structured in-depth interviews ranging from 35–60 minutes were conducted in private rooms in the clinic. Participants were interviewed in English or Afrikaans (a language widely spoken in the Western Cape). Interview schedules were available in both languages. All interviews were recorded, transcribed verbatim and those in Afrikaans translated into English. Ethics approval for the study, was granted by Stellenbosch University, Health Research Committee (reference number N15/08/077). Additional consent to conduct interviews was obtained from the hospital's administration.

**Table 1:** Summary of Participants' Details

Name*	Age	BC** stage	Diagnosed	Highest edn level	Used CAM	CAM Types
Ursula	35	2	2013	tertiary incomplete	Yes	Ointments [Vicks, Uncle Daan's & Olive wood]; Maizena
Denise	36	1	2016	matric/secondary complete	No	N/A
Emma	38	3	2010	matric/secondary complete	Yes	Homeopathy
Valerie	38	2	2012	secondary incomplete	Yes	Herbs [various cancer bush tea extracts]
Kayla	41	2A	2012	tertiary incomplete	No	N/A
Olivia	47	3	2003	secondary incomplete	Yes	Sutherlandia tablets
Teresa	47	4	2013	secondary incomplete	Yes	Herbs [buchu, bitterpatat, dassiepis, wynruit; ointment [camphor & other - unamed]
Stella	50	2	2015	secondary incomplete	Yes	Herbs [kanker bossie (sutherlandia), wynruit]; marijuana oil & seeds
Hannah	51	3B	2015	matric/secondary complete	Yes	Hydrotherapy; green diet
Natasha	53	1	2013	did not answer	Yes	Rooibos with cayenne pepper
Roline	53	1	2013	primary complete	Yes	Herbs [Krimitart & vaalbos]
Carol	53	Unknown	2009	tertiary incomplete	Yes	Tablets from alternative medicine practitioner
Francine	55	1	2010	matric/secondary complete	Yes	Somatology; aromatherapy; ozone
Pam	56	3	2014	no formal education	Yes	Maizena
Jaylee	63	2	2014	secondary incomplete	No	N/A
Betty	68	3	2014	tertiary complete/graduate	No	N/A
Greta	78	Unknown	2010	matric/secondary complete	No	N/A

*Pseudonyms; BC** =breast cancer

## Data analysis utilised IPA procedures

IPA data analysis process employs a double hermeneutic^21^,^22^. This double hermeneutic comprises a hermeneutic of empathy where the researcher attempts to engage with an individuals' lived experience at the descriptive level of meanings ascribed by individuals to their specific lived experiences as subjectively experienced and described, and a hermeneutic of questioning as the researcher engages critically in an attempt to make sense of individuals' meaning making^21^,^22^,^24^. In this study, where data was collected by several interviewers, making sense of data included regular meetings between the lead author and other interviewers to check that data collection was proceeding as planned, to discuss field-related challenges, and to seek clarity on contextual nuances. Data transcripts were analysed consecutively, with a focus on idiographic experiences, before considering themes across cases^21^,^22^. This entailed reading through each transcript severally while noting descriptive, linguistic, and conceptual comments and later, emerging themes^22^. IPA data analysis is an iterative and inductive process as researchers move back and forth between the hermeneutic of empathy and the hermeneutic of questioning as they engage with interview data, though one may also draw on previous literature and work in making sense themes emerging from primary data 22, 25. The double hermeneutic iterative process yielded emerging themes both within individual narrative accounts and, once this was done, themes arising across cases were identified. Initial data analysis at descriptive level, culminating in identification of preliminary emerging themes, was conducted by the lead author. Subsequent analysis and further theme development were conducted iteratively by both authors in consultation.

An audit trail containing all research activities and decisions at each stage of the study was maintained throughout the study to ensure dependability 26, 27. As it was not possible to engage in member checking due to the appointment system at the clinic which meant we could not follow up with patients outside of their clinic appointment times which were several months apart, measures to promote credibility included each interviewer taking observational fieldnotes immediately after each interview and discussing these with the lead researcher, including discussions on denotative versus connotative meanings in the context of translation of interviews from Afrikaans to English. To further boost credibility, all the interviewers documented reflexive accounts of their impressions, positionalities and how these shaped their interactions with participants, in consistency with IPA and other constructivist approaches that embrace subjectivity and focus on acknowledgement rather than denial of bias 26, 27. In addition, we sought to give as much contextual detail about the setting and research process as possible to facilitate transferability^26^,^27^. Dependability, credibility and transferability, contributed to confirmability of the research 26.

## Results

Of the 17 women included in the study sample, 12 utilised CAMs and of these, eight women spoke about disclosure during the interviews. In this section, we present three sub-themes features in participants' narrative accounts of CAM use. As noted in the introduction, discussions about CAM use featured in the context of broader descriptions of women's breast cancer experiences. The three sub-themes presented in subsequent paragraphs are situated in three overarching themes descriptive of their lived experiences of breast cancer: *the setting*, that is the physical public health facility setting, in which we present the sub-theme ‘doctor-patient interactions’; *patient perceptions and agency*, in the context of patient-doctor interactions, in which we present the sub-theme ‘superior knowledge’; *decision-making and prevailing circumstances*, where we present the sub-theme ‘contingent disclosure’. We discuss each in turn.

### Doctor-patient interactions: ‘...there's not a gap where I can tell my story’

Hannah, herself a nurse, recalled that none of her doctors were forthright about the possibility of cancer but instead used indirect communication, for example, ‘it's very bad’ or required her to report back to the hospital immediately.

She reported that breast cancer patients were ill prepared for chemotherapy:

**Hannah:** I felt that they should have prepared us for chemo; they did not prepare us. I came here for my follow up ... we were sitting in the waiting room, and the doctor came in and called out a lot of names and that day they said “you [sic] going over to Z Block...” So, when we went there and doctor said “this is Z Block, and you guys are the ones that need chemo because of the cancer that is big and it can spread, and you are starting today with your first...” And then everybody was shocked.

Following bad side effects of her first chemotherapy session, Hannah absconded from treatment and resorted to alternative natural remedies: green diet, hydrotherapy and exposing her breasts to direct sunlight every morning. Against this background, Hannah had reservations about disclosing her CAM use to the doctor:

**Interviewer:** And did you tell the doctors what you were doing?

**Hannah:** Um, to tell you the truth I did not, I did not [laughs]

**Interviewer:** What stopped you from telling them “Listen, I'm on this vegan diet. I have cut out this and I'm exercising and I'm using salt and hydrotherapy” What got in the way of saying “this is what I'm doing?”

**Hannah:** I think, like I said, the doctors were very helpful, but I see that there is not a gap where I can tell my story. It is always [what] they are doing, and you need to be happy and off you go. So, there was never that gap for me to tell them my story about what I'm doing, you know. At face value, Hannah's reason for non-disclosure of CAM use was a pragmatic one suggesting that, had there been opportunity, she would have discussed the matter. However, her response might also depict the systemic nature of a physician-centred approach in a public healthcare facility which may privilege what ‘they [doctors] are doing’ rather than focus on patient experience.

### Superior knowledge: ‘Only the doctor knows what to do’

Based on their personal experience coupled with others' testimonials about CAM use, Natasha and Roline seemed convinced about CAMs. Natasha shared the story of her late husband who was diagnosed with terminal cancer. Doctors expected him to survive for a month, but he lived for a year, which she attributed to drinking black rooibos tea with cayenne pepper daily from the point of diagnosis. Natasha, thus, took to diligently drinking rooibos and cayenne pepper daily alongside biomedical therapy and claimed that it helped with recovery after surgery. Roline explained that her mother gave her an herbal concoction comprising krimidit (cream of tartar), which she described as a cancer bush, and vaalbos (another bush). Though women like Natasha and Roline seemed persuaded that CAM contributed to their healing processes, they did not disclose CAM use to their doctors and/or were hesitant about recommending CAM to others:

**Interviewer:** Did you tell the doctor about the cayenne?

**Natasha:** No, I did not [laughs]...I have not told them. You may not use advice like that; you can only use the pills they prescribe.

**Interviewer:** What do you think he would have told you about it?

**Natasha:** the doctor will [silence] no, I don't know.

**Interviewer:** but you believe it works, right?

**Natasha:** It works. It works.

**Roline:** You can't give someone advice on cancer, what you should drink, because it's a different type of illness. Only the doctor knows what to do.

Ironically, the women who were convinced about the value of CAM still considered the doctor's advice superior as captured in Roline's assertion, “only the doctor knows what to do”. They may have perceived the doctors' advice as superior based on their professional expertise in contrast to their own beliefs in CAM, based on lay knowledge and testimonials. Perhaps their hesitation might be because CAM is not integrated into conventional public healthcare facilities thus, the women may be fear that their doctors may respond negatively to use of ‘other’ therapies.

One may tentatively infer that the emerging narrative is one of women straddling and cautiously navigating between two dichotomous worlds of biomedical and CAM therapies. This sense of two separate worlds was subtly conveyed in Olivia and Stella's accounts. Olivia was persuaded that CAM aided her healing process as well as that of a relative and friend who also cancer. However, she did not disclose this to her doctor:

**Interviewer:** I wanted to ask you about the sutherlandia; you didn't tell the doctor that you were taking it?

**Olivia:** No...I didn't need to. I don't think[so]...

Olivia, who took CAM simultaneously with biomedical therapy, did not consider information about her use of CAM relevant to share with the doctor.

Stella enthusiastically shared details about the role CAM played in cancer healing process during and after she completed her biomedical therapies:

**Stella:** They stopped my chemo and radiation. At the moment I don't know what is going on in my body but I drink my herbs...they [rastas] pick it in the mountains... kanker bossie [cancer bush]/ sutherlandia...the other one is wynruit...I also add aloe...we mix them together, draw it in water...I feel very good! You can feel how it moves through your body...they [rastas] say that the [marijuana] oil and the seed/pips are good for cancer...we crush it ... then drink that oil-water...I'm not getting any treatment at the moment. So, then I will just treat myself from home [laughs].

Though Stella acknowledged not knowing what was going on in her body, perhaps from a medical perspective, there was a sense of agency and pride in what she regarded as her superior knowledge which enabled her to self-treat with CAM and ‘feel very good’. For Stella, navigating between the two worlds was evident in the fact that she did not mention disclosing her CAM use to her doctor but had no reservations about recommending CAM to other patients:

**Interviewer:** And if you could give somebody else advice, what would you tell them?

**Stella:** I would tell them about the herbs; drink the herbs.

**Interviewer:** So, you have a great deal of faith in the herbs?

**Stella:** if they put me on the ‘year pill’ [tamoxifen] now, I will drink it but I will still drink my herbs every day for the rest of my life.

Likewise, she would adhere to biomedical treatment but would also continue with CAM treatment, suggesting that each type of treatment had a place in each of her worlds

### Contingent disclosure: ‘...that was the only reason’

Emma, who visited a homeopath and disclosed this to her doctor, explained that her doctor did not respond negatively to her disclosure:

**Interviewer:** Did you tell any of the doctors that you had been to see him [homeopath]?

**Emma:** Um... oh yes! yes sorry - yes, I did actually when I went to Dr X; I did tell her that I went to him [homeopath], because I was just worried and I just wanted to make sure and I wanted him to tell me I don't have cancer; that was the only reason...

**Interviewer:** And what was the doctor's response when you told her that you went to a homeopath?

**Emma:** Ag, no she was fine. Ja she was fine and it's not like she was, she feels [sic] less, because she is also a doctor. But the thing is for me, my concern is that I just wanted to hear something from another doctor. That was my only thing. Not that I think he [homeopath] will be better. Emma's impression that her doctor did not ‘feel less’ [inadequate] because she sought a second opinion introduces a comparative dimension: who is better between the doctor and homeopath? Emma's need to justify her visit to the homeopath, almost as though she was trying to reassure her medical doctor that her view was superior to the second opinion of the homeopath, subtly implies that doing so might be potentially problematic in the sense of questioning the doctor's authority. Emma did not appear to have had any reservations about informing the homeopath, whom she also referred to as a doctor, that she consulted with a clinician prior to seeing him. This is similar to Carol, who also referred to her CAM practitioner as a doctor and informed him of her visit to the physician but did not disclose CAM use to her doctor at the breast clinic to avoid conflict.

Francine's decision to tell her doctor about receiving somatology treatment from her daughter was motivated by physical discomfort and frustration:

**Interviewer:** Do they [doctors] know that you've gone to your daughter for lymph drainage?

**Francine:** No that was in Town Y.

**Interviewer:** Will you tell them that you're going this time?

**Francine:** Ja, I'm going to tell, because this is really quite frustrating...this arm is fine but she doesn't know how to do this one because she said she's not sure to which lymph she must drain it to[sic]. So, I just wanna ask them must I go for lymph drainage, and where do I go?

This excerpt demonstrates Francine's valuing both biomedical and complementary therapies, recognition of the limitations of her complementary [somatology] treatment and the relative superiority of biomedical professionals implied in her persuasion that they would know if/which lymph node(s) should be treated and where to get such treatment.

## Discussion

Most breast cancer patients continued to use CAM concurrently with biomedical treatment, with many opting not to disclose to their healthcare practitioners for various reasons. The healthcare setting influenced decisions to disclose CAM use or not. Findings suggest that the manner of doctor-patient communication coupled with existing hierarchies may deter patients' disclosure of CAM use. The public health care system in South Africa is overstretched 28, 29, and pathways from primary to tertiary care can be difficult 18. This is likely to impact the quality of service rendered, including inadequate information on treatment options and possible undignified treatment of patients 28, which is not unique within SSA contexts, as a joint Ugandan/South African study demonstrated 30.

Study results point a health care setting shaped by paternalism as a cultural norm, where health care professionals' expert views are privileged over lay patients' views on health matters in the sense that the doctor knows what is best 31. Paternalism may lead to fear of reproach if one discloses CAM use, resulting in clandestine use of CAM documented in studies of cancer patients SSA countries 32–34. In such instances, disclosure may either be considered a last resort, based on prevailing circumstances, or reserved for fellow patients who might benefit from CAM use. In this context, paternalism may bear the negative connotation of patronising in the sense of imposing of Western biomedical values on patients with little regard for their cultural values 35, for example, those that embrace CAM. Similar sentiments were echoed by South African health practitioners who endorsed medical pluralism but were reticent to disclose referring their patients for CAM services in fear of reproach from colleagues who subscribed solely to biomedical practice 14. However, paternalism may be preferred over patient autonomy in certain cultures and contexts 35, 36, for example, where patients have low levels of general and medical literacy 31.

Our results offer some perspective on patients' perceptions and agency with reference to CAM disclosure. It is notable that though breast cancer patients in the current study appeared disempowered within the biomedical system, they portrayed a sense of agency in decisions to disclose CAM use or not as they and negotiated mostly clandestinely between conventional therapies and CAMs. Sociocultural notions of holistic wellness seemed to guide decisions regarding medical pluralism while women simultaneously straddled between biomedical and CAM worlds, which resonates with findings of a study which investigated the interactions between traditional health practitioners [THPs] and biomedical practitioners caring for HIV/AIDS patients in Eastern and Southern Africa 14. It is possible that because CAM use operated at a more interpersonal level of family, friends and community networks, women had some agency in the process. They learnt how to ‘brew’ various concoctions, where to obtain ointments and where to find CAM practitioners. Information shared was laced with folk knowledge and cultural beliefs about effective remedies thus, possibly familiar, and easy to grasp.

Three implications for policy and practice may be drawn from this study. First, our study reinforces findings of previous studies which show CAM use in the context of cancer care and failure or reluctance of patients to disclose CAM use to biomedical practitioners. This is consequential in light of concerns raised by South African researchers about CAM use contributing to delays in help seeking 14, 18, interruptions to biomedical treatment regimens 20 and the possibility of negative drug reactions in the context of medical pluralism 16, 17. As custodians of a health care system that privileges the views of biomedical health professionals, doctors and other health care personnel should look for opportunities for initiating conversations with patients to encourage CAM disclosure in non-threatening ways. Second, against the background of South Africa's recognition of THPs and the need to foster collaborative practice between THPs and biomedical practitioners, our findings suggest that there is still work to be done to facilitate the integration of THPS and CAMS into the public health sector, so that patients feel at ease discussing CAM use with their physicians. Third, our findings call for further consideration of South Africa's *Batho Pele* [ ‘people first’] in public healthcare settings, which implies respectful, empathetic and fair treatment of patients 37. However, Batho Pele is aspirational: though doctors and others may agree with and want to promulgate the principles, the reality of an overstretched system may make this impossible. Nonetheless, open communication about CAM use has been associated with higher patient satisfaction and improved quality of life among cancer patients 9, 38. What can be done without extra resources is an active attempt to communicate to patients that practitioners are aware that patients may use a range of resources (such as CAM), which is their right, but (and) in order to provide the best medical care, it is better that patients disclose so practitioners can adapt prescribing and treatment accordingly, because of the possibility of drug interactions and other potentially negative effects.

This study comprised a small sample of breast cancer patients attending a breast clinic in one tertiary public hospital in the Western Cape. This limits generalisability of the findings to the wider population and to breast cancer patients in private healthcare. It is possible conducting interviews in rooms within the breast cancer clinic and hospital set up may have hindered freedom of expression by participants in fear that interviewers may report CAM use to the doctors, despite assurance of confidentiality during the informed consent process. Interviewers reported that patients seemed reluctant and/or anxious to speak about CAM use, while a few outrightly refused to comment on the topic. Disclosure of CAM use was one of several sub-topics in examining women's overall lived experiences of breast cancer and, as such, the matter was not explored in detail. This warrants further research specifically focused on disclosure of CAM use, and with a wider and more representative sample, to increase understanding of dynamics around such disclosure. Despite the limitations, this study highlights the politics of disclosure of CAM use and potential implications for the healthcare system.
